# Analyzing territory for the sustainable development of solar photovoltaic power using GIS databases

**DOI:** 10.1007/s10661-019-7871-8

**Published:** 2019-11-20

**Authors:** Inmaculada Guaita-Pradas, Inmaculada Marques-Perez, Aurea Gallego, Baldomero Segura

**Affiliations:** 1 Departamento de Economía y Ciencias Sociales, Universitat Politècnica de València, Valencia, Spain; 2 Departamento de Ingeniería Cartografica, Geodesia y Fotogrametria, Universitat Politècnica de València, Valencia, Spain

**Keywords:** Solar energy, Solar radiation, Solar farms, Grid connection

## Abstract

Solar energy generated by grid-connected photovoltaic (GCPV) systems is considered an important alternative electric energy source because of its clean energy production system, easy installation, and low operating and maintenance costs. This has led to it becoming more popular compared with other resources. However, finding optimal sites for the construction of solar farms is a complex task with many factors to be taken into account (environmental, social, legal and political, technical-economic, etc.), which classic site selection models do not address efficiently. There are few studies on the criteria that should be used when identifying sites for solar energy installations (large grid-connected photovoltaic systems which have more than 100 kWp of installed capacity). It is therefore essential to change the way site selection processes are approached and to seek new methodologies for location analysis. A geographic information system (GIS) is a tool which can provide an effective solution to this problem. Here, we combine legal, political, and environmental criteria, which include solar radiation intensity, local physical terrain, environment, and climate, as well as location criteria such as the distance from roads and the nearest power substations. Additionally, we use GIS data (time series of solar radiation, digital elevation models (DEM), land cover, and temperature) as further input parameters. Each individual site is assessed using a unique and cohesive approach to select the most appropriate locations for solar farm development in the Valencian Community, a Spanish region in the east of Spain.

## Introduction

Sources of renewable energy (RE) have become a necessary alternative with which to face the growing and essential energy demand in the twenty-first century. Over the last decade, solar energy has emerged among the different types of REs (biomass, wind, geothermal and solar) as one of the most promising RE sources to replace fossil fuels in meeting the world’s future energy needs (Trappey et al. 2016). The International Energy Agency (IEA) estimates that solar power could provide as much as 11% of the world’s electricity production in 2050. However, this depends on a large number of countries implementing incentive schemes to promote solar energy production in the following decades, so that investment costs decrease (Song et al. 2015).

From 2000 to 2016, the predominant RE source in Spain was wind power, followed by hydro and solar power (Red Eléctrica de España 2018). Due to the orography of the Iberian Peninsula, and its location in the Northern hemisphere (its latitude and longitude coordinates being 36° 00° N and 43° 47″ N), Spain enjoys favorable conditions for both small- and large-scale solar energy production through cutting edge technology. It is for this reason that our localization strategy analysis focuses on power stations using photovoltaic (PV) technology to produce energy.

There are numerous studies which review the most suitable technologies for these systems. Some of them focus on the materials used in the production of PVPs (photovoltaic panels) and other P such as voltage drops and network losses in the large-scale integration of PV power generation into electric distribution systems. Other studies explore the topologies used to interconnect PVPs with inverters (grid connectivity) and the technology developed for inverters in order to compare their efficiency, control, cost, and weight, and analyze future trends (Trappey et al. 2016; Yu et al. 2011; Nemet 2006). In contrast, there are few publications which review the criteria for assessing the land use potential for solar farm development (large grid-connected PV power plants which have more than 100 kWp of installed capacity).

Finding the best location on which to install a solar farm is vital for the sustainability and economic feasibility of such projects. Traditional plans for PVP investments have been somewhat arbitrary, mainly because planners of solar power plant projects have barely considered analyzing them at a regional scale. Furthermore, previous studies assessing the potential of PVP investments in one area usually only used solar energy maps (Wang et al. 2016; Xin-gang et al. 2015), or solar energy maps and technology (Chakraborty et al. 2015; Chakraborty et al. 2017) although this is currently changing. Capital outlay for energy production is known to depend largely on the abundance of the resource and the distance to the point of energy end use. The availability of solar resource varies considerably as follows: (i) on a global scale between climates zones; (ii) on a local scale due to variations in terrain orientation, slope, elevation, etc.; and (iii) depending on the orographic conditions. Consequently, if the sun is to be used as a source of energy, it is convenient to carry out an in-depth study on the basis of location criteria to find optimal sites for the installation of PV power plants.

Developing land use strategies is essential for the rational and sustainable use of non-renewable natural resources such as land, since they allow this resource to be used in a planned, sustainable, and appropriate way (Akıncı et al. 2013). Indeed, knowledge and assessment of the area are vital for territorial planning, which is a complex process, firstly, because highly diverse factors related to the object of planning must be taken into account, and secondly, because of the difficulty of carrying out a joint analysis of all these factors (Galacho-Jiménez and Ocaña-Ocaña 2006).

The literature has extensively examined site selection issues for different land sustainable uses:

- Irrigated agriculture: Akıncı et al. (2013) determine suitable lands for agricultural use in the Yusufeli district of Artvin (Turkey) using parameters such as great soil groups, land use capability classes and sub-classes, soil depth, slope aspect, elevation, erosion degree, and other soil properties; Ismail et al. (2012) produce soil suitability maps for different types of irrigation land uses and consider the following as land suitability criteria: slope gradient, soil properties like depth, erosion, drainage, infiltration rate, available water holding capacity, stoniness/rockiness, salinity, exchangeable sodium percent, and calcium carbonate content; Mendas and Delali (2012) use multi-criteria analysis to produce a map with the best pieces of land for sustainable agriculture (productive and profitable agriculture that protects the environment and that is socially equitable), their main criteria being drainage, permeability, potential of hydrogen, electrical conductivity, active limestone, cation exchange capacity, soil texture, soil useful depth, slope, availability of labor, and proximity to roads.
- Ecosystem services: Geneletti (2013) develops a set of metrics to compare land use policy scenarios and to analyze their effects on the provision of ecosystem services including water purification, soil retention, carbon sequestration, provision of habitat for species, and timber production. These authors consider criteria such as elevation, slope, aspect, soil type, erosion, land cover proximity to infrastructure and urban areas, and proximity to existing plantations.
- Landfills: Al-Ruzouq et al. (2018) take into account social, economic, and environmental aspects like the distance to main roads, residential areas, airports, city boundaries, and wells, in addition to other aspects like elevation, slope, geology, and hydrology to select the most appropriate locations for landfills; Afzali et al. (2014) also study the availability of suitable sites for waste disposal using the following criteria: groundwater depth, distance of water resources to surface, slope, permeability, faults, environmentally sensitive areas, distance to residential areas and main roads, land use, and floodplains; for Khodaparast et al. (2018), the main factors in selecting suitable locations for waste disposal include slope, distance from urban and rural areas, surface water protection, land use and the distance from access roads, power lines, wells, saline groundwater zones, fault zones, historical sites, and airports.
- Tourism: Cetin et al. (2018) determine natural parks’ sustainable tourism potential using as criteria accessibility and location in the region, soil structure, biology and ecology buildings, technical and social infrastructure, and legal framework.
- Wind farms: Simao et al. (2009) use 19 decision criteria for the strategic planning of wind farm sites estimating both the impacts that a wind farm would have on the neighborhood and those elements that determine the suitability of a particular site for wind energy production, including the annual average wind speed at the site and its proximity to large settlements; Cavazzi and Dutton (2016) estimate the costs of energy production from *offshore wind farms* taking into account water depth, distance from the nearest ports or grid connection points, annual average wind speed, potential array losses, turbine availability, operations and maintenance costs, and financial parameters such as discount rates and project lifetime.
- Industrial areas: Reisi et al. (2018) consider various criteria for the selection of industrial establishments such as transport and communications infrastructure, water supply, wastewater networks, land availability, construction costs, availability of trained workforce, labor costs, unemployment rate, rivers and water bodies, soil, slope, environmental pollution concentration, noise, and land use.
- Groundwater protection: Fagbohun (2018) identifies the following factors as influencing groundwater recharge: land use, lithology, drainage and lineament characteristics, slope, soil, and rainfall.

Goleiji et al. (2017), for their part, examine site vulnerability to natural threats and produce a forest fire risk map assessing factors such as slope gradient and aspect, altitude, land cover, distance to settlements and roads, wind speed, vegetation index, annual rainfall, and temperature.

As may be seen from the numerous references, there are many studies on soil and location suitability for different uses and activities.

Renewable energy strategies for sustainable development, particularly solar energy planning, require studying both the impact of PVPs at regional and local levels and the suitability of the areas for solar farm development (Chakraborty et al. 2017). Moreover, an important part of the solar energy planning process is to determine the factors or criteria that affect the availability of this resource. It is therefore essential to consider all aspects (technological, economic, and environmental), since it is a complex process which needs complete information about a wide range of criteria and their implications for deciding on the available areas.

Topographical and soil features are among the most relevant factors for solar energy planning. Their analysis involves a large variety and amount of physiographic data, which include climate characteristics such as solar radiation; internal soil characteristics such as natural fertility, depth, texture, and geology; and external soil characteristics such as slope, land cover, erosion, orientation, elevation, flood risk, and accessibility.

Currently, there are approaches available, based on geographical information systems (GISs) and spatial analysis, which can provide considerable support to management and decision-making processes (Mendas and Delali 2012). These tools make it possible to interpret spatial components and site information for solar farm land use and any technology chosen.

In our study, the criteria for deciding on one location or another are defined by examining the literature on accessibility (Janke 2010; Gastli and Charabi 2010), grid connection (Janke 2010), orientation and slope (Janke 2010; Gastli and Charabi 2010; Sánchez-Lozano et al. 2016), land cover (Wang et al. 2016; Janke 2010), and latitude and longitude (Jo and Otanicar 2011). Sánchez-Lozano et al. (2016) introduce criteria to evaluate jointly the following aspects: soil properties, slope, orientation, grid connection, solar radiation, and temperature. Based on this review, we synthesized all the information, chose the criteria, and then defined a preliminary list. This list of criteria was submitted for approval by a group of experts on solar PV farms. Based on their assessment, we established the final list of criteria for the site selection of PV farms (see Fig. 3).

However, according to the literature reviewed, there are some knowledge gaps associated with solar farm site selection. Firstly, the literature considers only a limited number of variables and does not distinguish the excluding aspects that make solar farm projects unfeasible for certain areas (risk areas, legal exclusion, etc.) from those aspects that simply limit or condition the activity (slopes, orientation, temperature, radiation, etc.). Secondly, technology criteria are not taken into account when in fact, depending on the type of technology chosen, solar farm activity may be limited to a greater or lesser degree in certain locations (Maleki et al. 2017). Thirdly, grouping criteria might not be possible due to the small number of variables used, i.e., the criteria considered may not allow us to identify the most advantageous areas. For instance, the distance to transmission lines could emerge as an important factor when comparing areas with abundant solar energy resources at local and regional levels and when the values of solar radiation are similar in all the area studied (Yushchenko et al. 2018).

Thus, although our methodology is developed based on the aforementioned literature review, an alternative approach is proposed for treating the solar planning criteria. The analysis is carried out in two stages: first, certain areas are excluded, which simplifies the subsequent analysis and allows for further information to be included; and secondly, the aspects to be considered when selecting locations are examined, and classified into criteria and sub-criteria in order to carry out more detailed partial analyses using specific criteria.

This two-step approach combines legal, political, and environmental aspects with geographic and technical-environmental criteria, which include solar radiation intensity, local physical terrain, environment, climate, location, and the distance to roads and the nearest power substations. Additionally, we use GIS data (time series of solar radiation, digital elevation model (DEM), land cover, and temperature) as further input parameters. Each individual site is assessed using a unique and cohesive approach to select the most appropriate locations for solar farm development in the Valencian Community, a Spanish region in the east of Spain. Our aim is to provide an innovative system for spatial planning problems by creating an argumentation map to analyze the different factors that determine the areas not excluded.

The objective of this study is thus to provide a methodology with which to identify potential PV power generation sites in a specific area and thereby support the development of new PV power stations as well as strategic RE planning. Moreover, the results of our study can help find potential areas for solar farm development in any country.

Further research might include other criteria like building costs and local demand which can also affect site selection. They could be applied in further steps to assess the candidate locations and select the suitable areas (see Fig. 3), but these criteria are specific to particular farm projects. Our research work here stops at the second level of analysis.

This paper is divided into the following sections: after this introduction, information is provided on the area of study and the parameters used in the solar farm site analysis. Next, there is a description of the GIS tool used for data criteria management, as well as its integration for the area selection. This is followed by the results and discussion. The final section presents the conclusions together with some final comments.

## Area of study

**Fig. 1:**
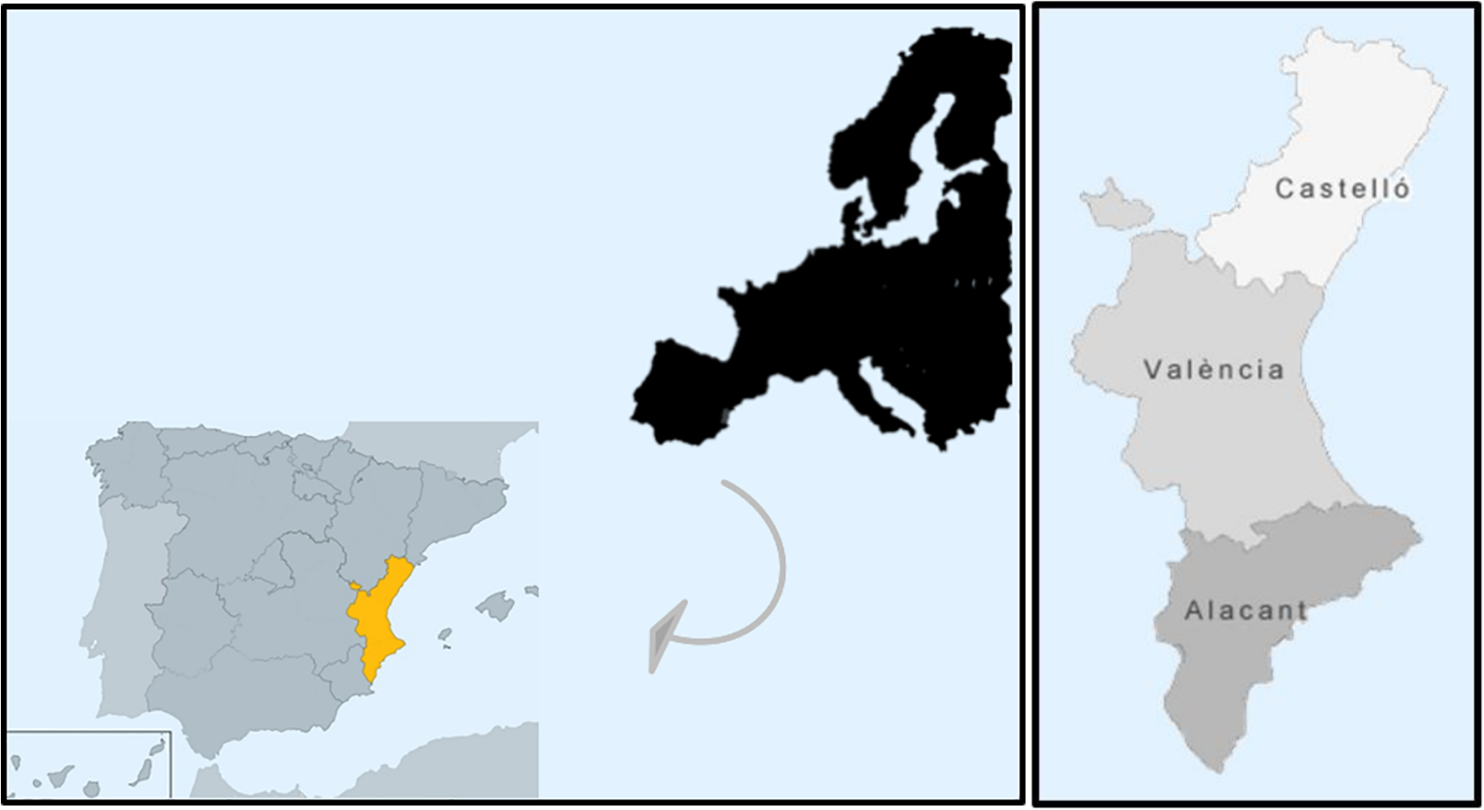
Study area of the Valencian Community in Spain

We apply the proposed GIS-based approach to select locations for the installation of solar power plants in the Valencian Community, a Spanish region in the east of Spain (Fig. 1).

In 2017, there were approximately 5 million inhabitants residing in this area, which represents 10.5% of the total population in Spain and occupies the second position in terms of population density (more than 210 inhabitants/km^2^)^1^. The Valencian Community includes Castelló, Valencia, and Alacant. It is an economically developed region, covering more than 23,000 km^2^, and located in what is known as the Mediterranean Corridor, a relevant economic area at the European level. Its current energy consumption stands at 23,000 GWh in 2018 and fossil fuels are the main source of energy (66%), with RE sources having barely been implemented. In fact, the Valencian Community is a region that consumes more energy than it produces, with a negative balance of approximately more than 2 GWh.

The aim of the Valencian Government is to give priority to RE sources rather than conventional ones, promoting actions that intensify their contribution to energy production in the region at the expense of fossil fuels. Although the main objective is to reach at least a 16% share of renewable energy in gross final energy consumption, the final goal is to achieve a greater use of REs. The orographic conditions of the Valencian Community are propitious for the installation of new PV power plants, which would help reach the proposed objective. The Valencian Institute of Cartography, supported by the Valencian Government, has an important set of maps^2^ available that provide comprehensive information on relevant spatial criteria for site selection, showing environmental protected areas, and high mountain and forest areas, as well as suitable land for agriculture. In addition, there are maps with infrastructure data containing the high- and low-voltage power lines of the Valencian Community. Maps from the Spanish National Geographic Institute were also used in our study. Solar radiation data was obtained from the European Commission PVGIS (Photovoltaic Geographical Information System) website. All this information was used to select the most suitable sites in the region for the construction of PV power plants.

**Fig. 2:**
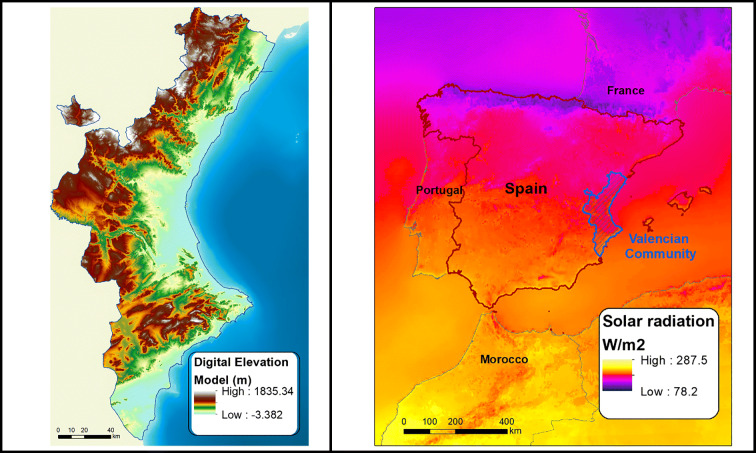
DEM and solar radiation in the Valencian Community

In the Valencian Community, the elevation declines gradually from west to east. The maximum altitude is over 1800 m, and solar radiation is from a minimum of 179.3 W/m^2^ to a maximum of 219.7 W/m^2^. The highest solar energy densities occur in southern areas with more than 200 W/m^2^. Given the great availability of solar energy resources, the Valencian Community has the potential to develop PV power stations (Fig. 2).

## Methodology

### Land use criteria for solar farm analysis

**Fig. 3:**
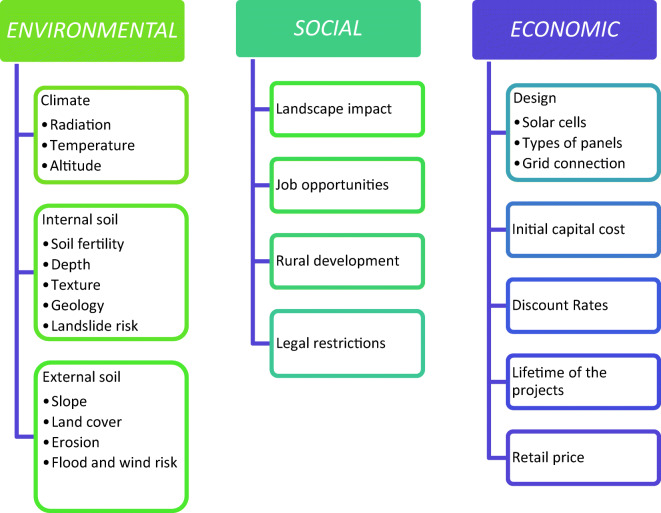
Main criteria used in the site selection model for PV power plants

There is a considerable amount of different criteria to be taken into account in the installation of solar power plants. This makes solar energy planning increasingly complex. The criteria can be grouped into three categories: environmental, social, and technical-economic (Fig. 3). Within the *environmental criteria*, aspects related to climate and soil are considered:

- Climatic criteria: temperature, altitude, and radiation.
- Internal soil characteristics: natural fertility, depth, texture, and geology.
- External soil characteristics: slope, land cover, and erosion.

Regarding the Valencian Community, there are well-defined legal restrictions for soil use. The following laws related to preserving areas of ecological value have been approved:

- Law 11/1994 of December 27 on Protected Natural Areas in the Valencian Community which establishes Nature Parks as protected areas. This law expressly prohibits any action involving substantial transformation of the physical or biological environment. It also limits any actions that can hinder accessibility to protected natural areas.
- Law 10/2004 of December 9 of the Valencian Community on protected undeveloped land which expressly prohibits the building of installations that are not compatible with the use, conservation and care of natural resources in protected areas.
- Law 42/2007 of December 13 on Natural Heritage and Biodiversity which establishes the obligation of the public administration to prevent the deterioration of the natural heritage.
- Law 5/2014, of July 25 on Territorial, Urban and Landscape Planning of the Valencian Community which regulates the building of PV farms within the view of or affecting the view of valuable landscapes.
- The Natura 2000 Network of the Valencian Community takes into account the Community Directives, i.e., Directives 92/43/EC and 2009/147/EC, which are aimed at the conservation of natural areas and biodiversity. In the Natura 2000 Network, the Special Areas of Conservation (SAC) and Birdlife Special Protection Areas are also included.

Among the social criteria that must be considered are those aspects that can influence the social value of PV power plants:

- Landscape impact
- Job opportunities
- Rural development

Finally, the technical and economic criteria are determined partly by the technological advancements and partly by the investment parameters for solar photovoltaic development. The technical and economic criteria are analyzed together since certain aspects determine others:

- Design: solar cells, type of PV panels, and grid connectivity
- Initial capital cost
- Discount rates
- Lifetime of the projects
- The electricity retail price which determines the profitability of solar farms

**Fig. 4:**
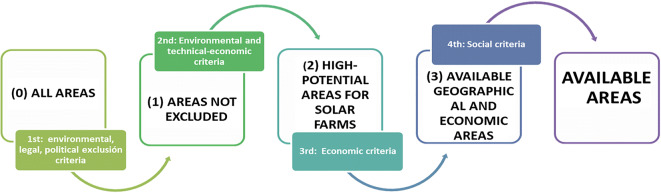
Modelling to select available sites for PV farms

All the above-described criteria are used throughout the four stages (levels) of the decision-making process (see Fig. 4).

The first level of decision is determined by the political and legal conditions which establish whether a solar power plant can be installed or not. These conditions are set by the economic policies and environmental regulations made by the government of the region under study. In our paper, we consider those established by the Spanish government and the Valencian regional government, according to the division of competences set out in the Spanish Constitution. The reason for using legal exclusion criteria is that, for example, in the case of land classified as urban only houses or factories can be built there, while solar parks cannot be installed (Law 10,^2004^). Other areas which must be excluded are nature reserves, national parks, natural monuments, and other environmentally sensitive areas, which are protected due to their natural outstanding or unique value, as well as historical and tourist monument sites (Law 42,^2007^), and rivers (Janke 2010; Gastli and Charabi 2010; Wang et al. 2016). Other areas that have to be removed in our study are those included in the Natura 2000 network, which are also considered nature reserves: Sites of Community Importance (SCI), Birdlife Special Protection Areas, as well as cultural heritage sites (Law 10,^2004^). PV installations can affect the movement of migratory birds and cause deforestation and the creation of physical barriers which damage wildlife, etc. (Sanchez-Lozano et al. 2016).

Other environmental criteria which lead to exclusion are areas with risks involved, such as floods, landslides, and windy areas, or with geological characteristics that make the area unsuitable for installing PVPs.

Once the *areas not excluded* are identified, we step into the second decision level in which geographical location criteria are used. Solar radiation is the main and decisive factor (Gastli and Charabi 2010; Janke 2010) at this level. In the case of the Valencian Community, solar radiation is above the minimum level required (more than 5 h/day: min 179.3 W/m^2^ to max 219.7 W/m^2^).

In order to make the construction of the solar farm easier and taking into view possible repair and maintenance issues, those sites closer to roads are considered to be more suitable (Gastli and Charabi 2010; Janke 2010). The distance to the nearest electricity network is also a determinant factor (Wang et al. 2016). Difficulty in connecting PV-generated electricity to the grid depends on the location of the site. The transmitting distance to grid connections (substations) is also essential, since power losses make it harder to achieve a real supply of RE electricity (Song et al. 2015).

Other site features, such as land slope, should be taken into consideration, i.e., those sites with a steep gradient where it is difficult to install solar panels should be excluded. Gastli and Charabi (2010) recommend flat terrain or gentle slopes facing south with less than a 5% graded slope for large-scale PV power plants. According to Wang et al. (2016), fields flatter than 5° are a technical requirement to ensure that all the PV modules can be installed at an optimal tilt angle. For Chiabrando et al. (2009), there is an optimal module tilt which depends on the latitude (at 46° north latitude this can be found between 30° and 35° if the time period of the analysis is 1 year), and for a case study they analyze a medium sloped hill of 20°. The orientation of the panel is another factor which affects the output of PV modules. The optimal module orientation is always south (or north in the southern hemisphere).

According to Janke (2010), the ideal locations for solar farms in terms of land cover are those with short vegetation, such as shrubs, prairie, grasses, scrub, steppe, agriculture, logged areas, or barren lands, which would not reduce solar insolation. Areas that are not suitable are those with sparse but taller vegetation (pine and juniper trees) or wetlands, due to their ecologic importance. Non-ideal areas are those whose land cover contains pine, subalpine, and aspen forests or areas on which it would be difficult to develop solar projects because of their inaccessibility or instability, or those areas which are not already developed. Areas with dunes, bedrock and scree, ice, cliffs, canyons, alpine tundra, and mines, as well as developed areas, also fall within this category.

Altitude and temperature are also factors that condition the normal functioning of solar panels and the production of solar energy. High altitude increases the difficulty of building PV power plants and can affect the transmission facilities. The highest solar energy densities occur in desert areas with high altitude and dry climate. Altitudes higher than 5800 m are not recommended, although it depends on the study area (Wang et al. 2016). According to Skoplaki et al. (2008), areas with average temperatures below 10° and above 20° should be excluded.

Table 1 shows the different criteria and the factors that affect the installation of PVPs and that can be directly or indirectly quantified. The criteria have been grouped according to their decision level. The selection column shows how each criterion or sub-criterion has been considered to select the locations.

**Table 1:** Criteria for the exclusion of areas and to identify high-potential locations for solar power plant

Criteria	Definition	Selection
Areas excluded		
Environmental exclusion		
Risk areas	Areas with landslide risk, specific detachments or flood risk	Areas without risks
Geology	Geology: lithology is an important factor when excavation works have to be carried out for the installation of PV farms	Calcareous rock areas are eliminated
Legal and political exclusion		
Urban land use classification	Areas where it is impossible to implement solar PV power plants because it is prohibited by urban land use classification	Residential, industrial, protected, public, public domain urban soil and military areas are eliminated
Protected natural areas	Areas included in the Natura 2000 network, or areas which have a special natural interest	The Natura 2000 Network areas are eliminated
High-potential areas for solar farm development		
Location criteria		
Solar radiation	Electromagnetic radiation emitted by the sun	Areas with radiation below 4.2 h/day and more than 5.0 h/day are eliminated
Grid connection	Distance to the nearest electricity network connection, to the nearest power line or power transformer substation	Areas more than 1500 m away from a grid connection are eliminated
Accessibility	Accessibility of the solar farm site to the nearest road (minimum distance in meters)	Areas with no access to local or district roads are eliminated
External soil criteria		
Slope and orientation	Physiography/level curves	Areas with a gradient higher than 15%, or with north/west orientation, are eliminated
Land cover	Consultation of/Information on the Spanish Land Cover and Use Information System (SIOSE 2015)	We identified the areas classified as suitable for agricultural and livestock production, rice cultivation, citrus and other fruit trees, olive and other woody crops, arable farming, with deciduous, coniferous, and evergreen species grassland, electric power production, as well as scrubland, idle land, and bare soil land. Areas with a cover below 30% are selected
Location criteria		
Temperature	Average temperatures measured by the weather station network of the Spanish Meteorological Service (AEMET)	Areas with temperatures below 10° C and above 20° C are eliminated
Altitude	Contour line map	Areas higher than 1800 m are eliminated

In future studies, economic criteria could be applied to these areas to identify the *available geographical and potential economic areas*. This is the third decision level. Finally, those areas defined as geographically and economically interesting should undergo a public participation process (fourth decision level), so that the society or population affected can decide what the best locations are in order to protect high-value landscapes, generate job opportunities, and foster rural development. As can be seen in Fig. 4, there are four decision levels, but our study stops at the second level.

Bearing in mind the large number of parameters and the considerations regarding optimal and limit values, etc., the use of a GIS is essential for all the information to be integrated, organized, and processed, as well as for the territorial variables to be linked together with other variables. Consequently, all the data must be georeferenced.

### GIS

As mentioned above, the selection of high-potential locations for solar energy production requires the use of a GIS in its different stages. With the help of this tool, geographical data (criteria maps) can be transformed and combined to obtain adequate and useful information for decision making in the spatial arrangement and distribution of activities, identifying suitable locations for these activities, here in particular the installation of solar power plants (Fung and Wong 2007; Ocaña-Ocaña and Galacho-Jiménez 2002).

Two types of data models have been used in this work: raster and vector models. Raster data models are represented by a grid of pixels, where each one stores information including their geographical location. In vector data models, geographical objects are represented by vector features, which maintain the geometric properties of the objects. Vector features are more suitable for representing boundaries and other geometric spatial objects. They take the form of points, lines, and polygons, which store information on their properties in the database.

This process involves the acquisition and management of spatial data, the performance of spatial analysis, and the creation of map-based outputs (Simao et al. 2009). GIS allows the information to be separated into various layers of themes and for them to be stored independently so that this information can be worked with easily and quickly. This also facilitates the possibility of relating existing information through the topology of objects in order to generate other information which could not be obtained otherwise. Geographical data is available from the different sources listed in Table 2, which also provides information on the available format, the scale used, and the publication date of the sources.

**Table 2:** Georeferenced data layers

	Data	Format	Scale	Publication date
Green infrastructure*	Influence areas of wetlands in the VC	Shapefile (vector format)	1/5000	2002
	Wetlands of international importance in the VC		1/5000	2002
	SAC in the VC		1/25.000	2013
	Birdlife Special Protection Areas in the VC		1/10.000	1992 (updated in 2009 with 25 new areas)
	Natural monuments buffer zones in the VC		1/5000	2007
	Nature reserves in the VC		1/5000	2011
	Protected landscapes in the VC		1/5000	2007
	Nature parks in the VC		1/5000	2007
	Local nature sites in the VC		1/5000	2002
	Sites of community importance in the VC		1/50.000	2001
Official cartography of the Valencian Community**	Transport network	Shapefile (vector format)	1/5000	2014
	Services and facilities			
	Administrative boundaries			
Urban planning***	Urban classification	Shapefile (vector format)	1/25.000	2018
Risk cartography***	Flood risk. Territorial Flood Risk Prevention Plan (PATRICOVA)	Shapefile (vector format)	1/25.000	2015
	Landslide risk		1/50.000	1991
Digital elevation model**	5m-resolution DEM in the VC	Raster	------	2010
Photovoltaic geographical information system	Solar radiation databases: average radiation both day and night measured in W/m^2^	Raster	------	2005–2015
SIOSE	Spanish Land Cover and Use Information System	Shapefile (vector format)	1/25.000	2011

*VC* Valencian Community, *SAC* Special Areas of Conservation

*Conselleria d’Agricultura, Medi Ambient, Canvi Climàtic i Desenvolupament Rural - Generalitat Valenciana

**Institut Cartogràfic Valencià

***Conselleria d’Habitatge, Obres Públiques i Vertebració del Territori – Generalitat Valenciana

As can be seen from the table, various cartographic sources have been used, such as maps from the Valencian Institute of Cartography (ICV), the National Geographic Institute (IGN), and the current Regional Department of Housing, Public Works and Territorial Planning, as well as a collection of thematic layers developed by the former Regional Department of Public Works, Urban Planning and Transportation. The solar radiation layer and the temperatures were obtained from the PVGIS website of the European Commission and from the Spanish Meteorological Service (AEMET), respectively.

**Fig. 5:**
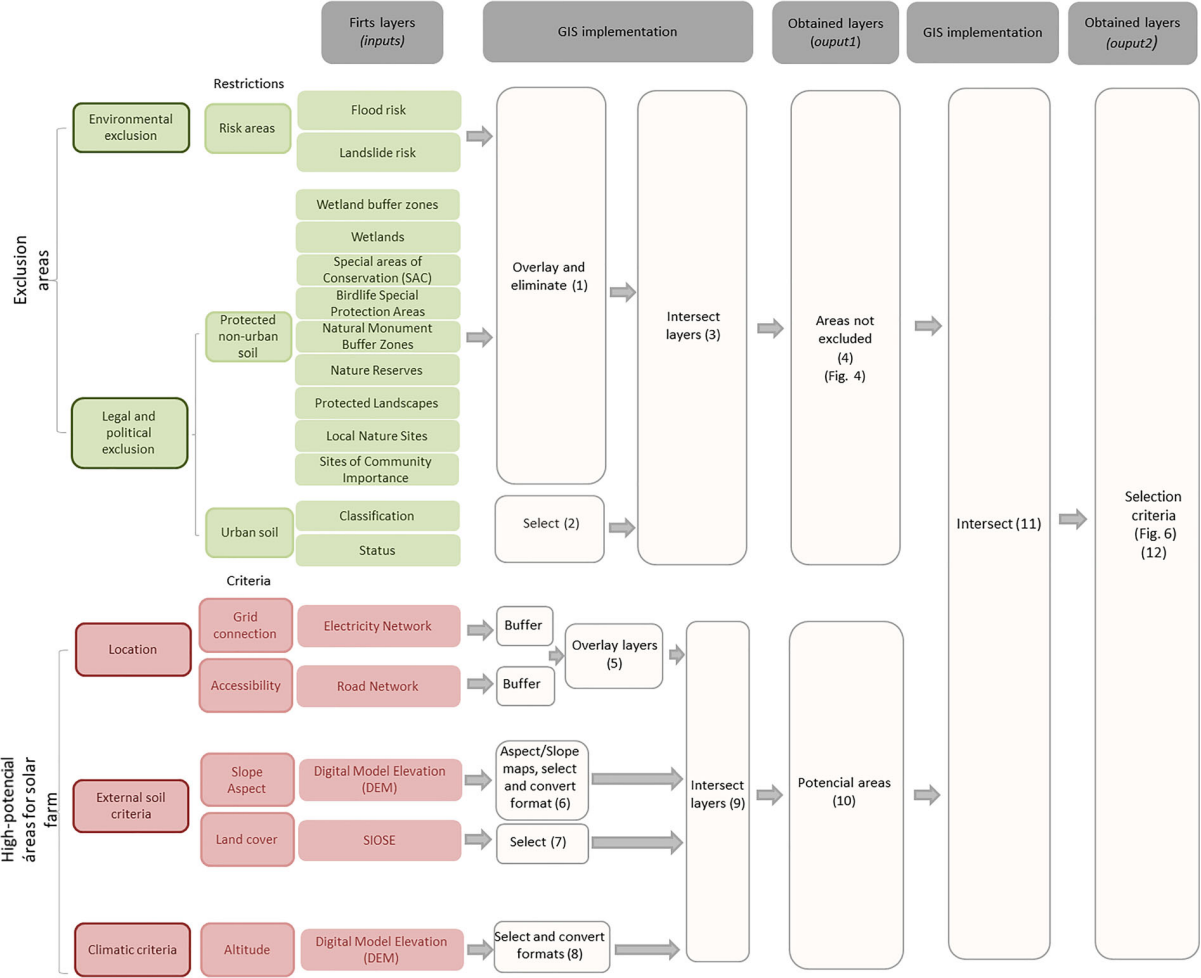
Methodology used for the site selection

Figure 5 contains a flow diagram showing the GIS-based methodology used in the whole site selection process, in other words, the steps taken in creating the layers. Protected area restrictions (protected non-urban and urban soils) or restrictions due to risks were introduced in the first layers (12 restrictions). These were overlaid to obtain the map of restricted areas, which were eliminated from the total study surface (Valencian Community layer). As a result, we obtained the areas not excluded. Environmental and technical restrictions were then introduced (4 restrictions). Next, the layers containing the external soil criteria (slope, orientation, and land cover) were overlaid and their distance to specific elements in the location criterion layers was calculated. In this step, the layer containing the areas with favorable distances was obtained. Finally, we intersected this layer with the not excluded areas layer to determine the areas with high potential for solar farms.

## Results

**Fig. 6:**
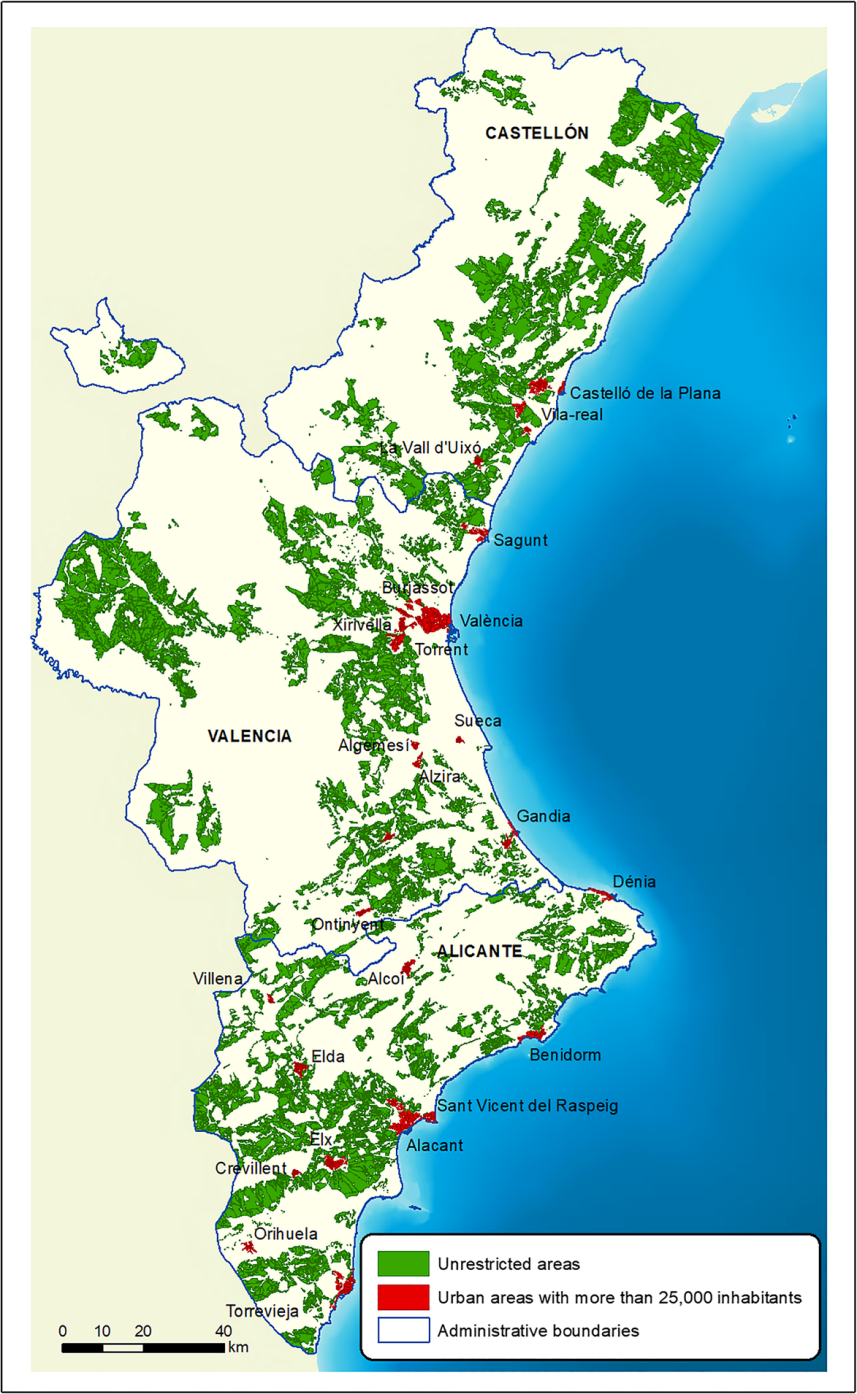
Areas not excluded

To be able to establish the not protected areas and those with no risks, the *green infrastructure* and the *risk cartography* layers were overlaid and the protected and risk areas were eliminated from the total study area (1). The next stage was to intersect (3) the resulting layer from the previous operation (1) with the layer containing the urban planning information, where previously only those areas classified as *common non-developable land* (2) were selected. In this way, the areas were obtained which could not be excluded for environmental or legal reasons, i.e., the *areas not excluded* (4). Figure 6 shows these areas highlighted in green.

**Fig. 7:**
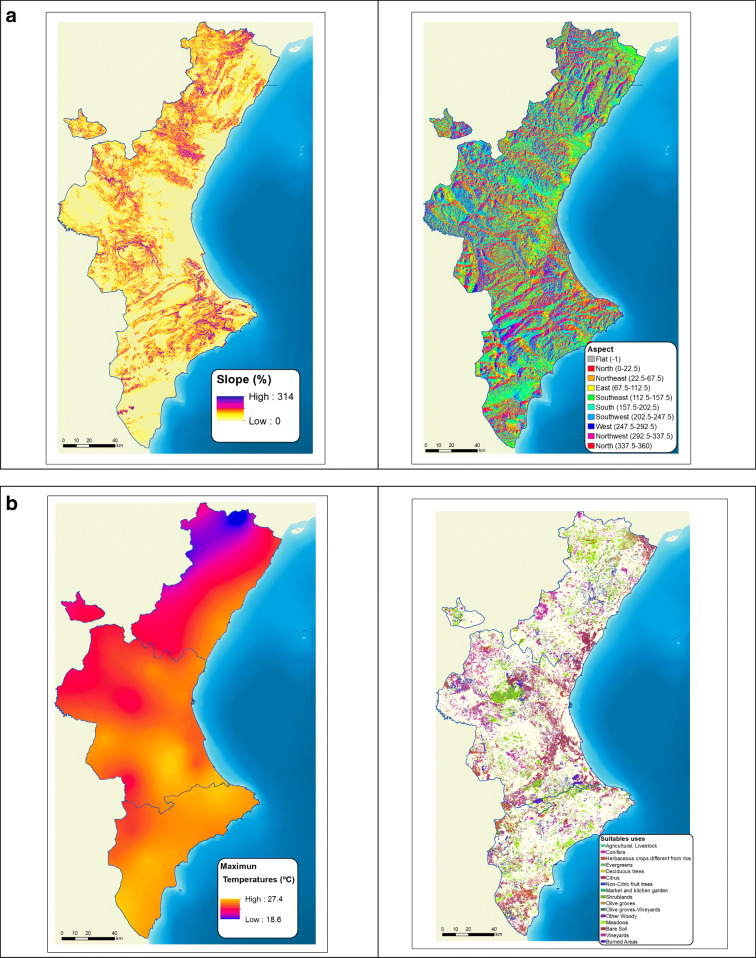
Selection criteria

A slope map and an orientation map were then created using a *digital elevation model* (Fig. 7a). With these two variables, the areas with the best topographic conditions were identified and the necessary format conversions (6) were then performed. Taking into account the recommendations of various studies cited above, the areas with northwest orientation and those with a gradient higher than 15% were removed (Fig. 7a).

The maps of the Spanish Land Cover and Use Information System (SIOSE 2015) enabled us to select the areas classified as having a land cover compatible with the installation of solar power plants (7). An analysis was carried out on land with soil classified as suitable for agricultural and livestock production, rice cultivation, growing citrus and other fruit trees, olive and other woody crops, arable farming, grassland, deciduous, coniferous and evergreen species, and electric power production, as well as scrubland, idle land, and bare soil land. Areas with a cover below 30% are selected (Fig. 7b).

Another topographic restriction is altitude. Taking into account that the maximum altitude of the Valencian Community is slightly higher than 1800 m, most solar analysis studies on this region select land with gentle slopes and no altitude limit. However, as the whole study area meets the criterion of an average temperature between 10° and 20° C, the only climatic factor considered was altitude. Using a DEM, we obtained those areas that met the established conditions and converted the raster format into vector format (8).

Next, we intersected (9) the layers containing the areas of influence of the electricity and road networks (5), the areas with suitable slope and orientation conditions (6), and land use/cover (7) and altitude information (8) thereby obtaining the most suitable sites for PV power plants.

**Fig. 8:**
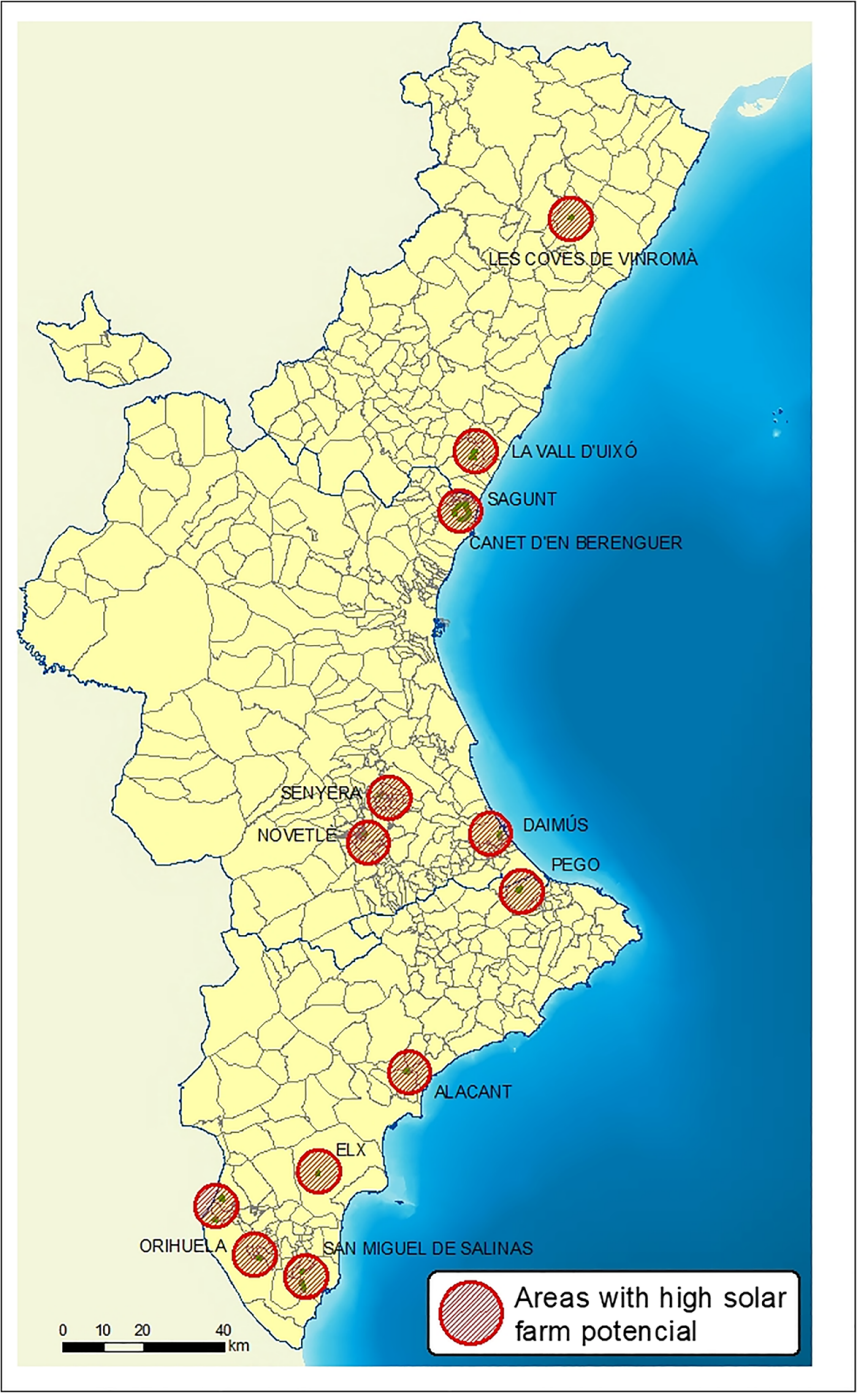
High-potential areas for solar farms

Finally, we intersected (11) the layer with the potential areas (10) with the layer containing the areas with no environmental or legal restrictions (4). The result was a total of 22 areas that met all the established conditions and criteria (12) (Fig. 8).

The different areas are considered spatial objects, i.e., polygons in the vector model and grid cells in the raster model, and constitute location alternatives. In this final step, the layer which identifies the *high potential areas for solar farm development* is obtained. Figure 8 shows these areas.

These potential locations should be assessed according to solar technology requirements so that economic criteria can also be considered. Their surface is between 10 and 50 ha and most of these areas are located in the province of Alicante.

## Conclusions

Assessing the different location alternatives in planning is a preliminary and decisive step when creating maps with suitable and economically viable locations for the usage of specific technologies.

For the sustainable development of a region, it is extremely beneficial to identify areas of land for solar PV power development for the following reasons: (1) transmission planning is optimized; (2) master plans for solar energy production can be developed; (3) it provides timely access to the selected land; (4) environmentally sensitive lands can be identified; and (5) it provides certainty for the solar power market (Gastli and Charabi 2010). The results of the present study can help governments with the planning of solar energy production and the implementation of PV power plants.

In this study, the spatial analysis began with defining the criteria with which to select suitable sites for solar farm development. The selection of criteria was based on the available literature on this subject: essential criteria enforced by government legislation and expert opinions on solar farm performance. The chosen criteria were then classified into three categories: technical-economic, environmental, and social. These criteria were grouped according to four decision levels and consequently four steps or phases in the selection process.

The first step was to disregard the areas with environmental, legal, and political restrictions in order to identify the *areas not excluded* for these reasons. Next, using GISs for these areas, technical and environmental criteria were applied to identify the areas with high potential for solar power plants. To carry out this process, it was necessary to find adequate thematic layers and cartography concerning the exclusion criteria.

The above-described methodology is valid for any area or zone in the world, but with some exceptions. On the one hand, it is important to bear in mind that the environmental, legal, and political criteria that determine the areas not excluded are specific to each country, and within each country they depend on the regional and local regulations for urban and territorial planning. The criteria for selecting areas with high potential for solar power development are the same everywhere, and it is highly probable that they have the same importance. However, it is recommendable to identify the stakeholders in each country or region and to conduct a survey with them to define the importance of each criterion, since a different weight can be given to each one of them. In the case of our study, we profited from an important geographic database available from the Valencian government, which allowed us to carry out a more accurate study and make the results more reliable.

It should be pointed out that the final outcome of 22 alternative locations does not provide a comparison of their suitability in relation to the others, both in technical and environmental terms as well as in economic terms. Site selection involves screening a large geographic area to select a limited number of suitable locations (alternatives). Alternative locations identified by the screening must be assessed further which will lead to finding the most suitable site among all alternatives.

In future studies, it would be advisable to use additional information and seek out expert opinions to create a ranking of suitable areas. It would also be interesting to incorporate the economic information of the selected areas to be able to identify the *geographically and economically suitable locations*, which would be the third decision level. And finally, using these areas as an object of further analysis, a public participation process should be undertaken (fourth decision level), whereby the society or population of the area in question can decide what the best locations are in order to protect high-value landscapes, generate job opportunities, and foster rural development.

## Footnotes

1 From the Statistical Site of the Valencian Government: http://www.pegv.gva.es/es.

2 See http://www.icv.gva.es/ca.
